# Three-Dimensional Planning for Vascularized Bone Grafts: Implementation and Surgical Application for Complex Bone Reconstruction in the Hand and Forearm

**DOI:** 10.3390/jcm14020440

**Published:** 2025-01-11

**Authors:** Maximilian Zaussinger, Karl Schwaiger, Jürgen Schwarzbauer, Kathrin Bachleitner, Matthias Holzbauer, Gudrun Ehebruster, Manfred Schmidt

**Affiliations:** 1 Department of Plastic, Aesthetic and Reconstructive Surgery, Kepler University Hospital Linz, Krankenhausstrasse 9, 4020 Linz, Austria; m.zaussinger@hotmail.com (M.Z.); gudrun.ehebruster@kepleruniklinikum.at (G.E.); 2 Medical Faculty, Johannes Kepler University Linz, Altenberger Straße 69, 4040 Linz, Austria; maximilian.zaussinger@kepleruniklinikum.at (J.S.); matthias.holzbauer@kepleruniklinikum.at (M.H.); 3 Doctoral Degree Program Medical Science (Ph.D.), Paracelsus Medical University, Strubergasse 21, 5020 Salzburg, Austria; karl.schwaiger@bbsalz.at; 4 Department of Plastic, Aesthetic and Reconstructive Surgery, Hospital of the Barmherzige Brüder Salzburg, Kajetanerplatz 1, 5010 Salzburg, Austria; 5 Department of Traumasurgery and Sports Traumatology, Kepler University Hospital, Altenbergerstrasse 69, 4040 Linz, Austria

**Keywords:** 3D technology, vascularized bone grafts, free MFC flap, bone reconstruction

## Abstract

**Background/Objectives:** Vascularized bone grafts have been successfully established for complex bone defects. The integration of three-dimensional (3D) simulation and printing technology may aid in more precise surgical planning and intraoperative bone shaping. The purpose of the present study was to describe the implementation and surgical application of this innovative technology for bone reconstruction. **Methods:** This prospective pilot study was conducted between June 2019 and June 2024. For this evaluation, patients who received vascularized bone reconstruction assisted with 3D technology were included. For reconstruction, the free medial femoral condyle (MFC) flap was used as the vascularized bone graft. Patient-specific 3D-printed templates, based on individual 3D simulations according to defect characteristics, were used for surgical planning, including flap elevation, shaping and inset. **Results:** A total of six patients (five male) with an average age of 39 years (range 19–62 years) and a mean follow-up time of 15 months (range 5–24 months) were analysed. The indications were as follows: avascular necrosis of the carpal bones, a metacarpal defect after tumor resection and pseudoarthrosis after a fractured ulna. Three patients received an osteochondral and three patients received a cortico-cancellous MFC flap. **Conclusions:** Our evaluation of clinical application revealed enhanced preoperative planning as well as intraoperative performance. Although the implementation for this technology is challenging, the new insights gained in planning and surgical guidance have led us to incorporate this technology into our standard routine.

## 1. Introduction

Vascularized bone grafts have been introduced as a promising treatment option for the reconstruction of bone defects [1]. These grafts, harvested with their own blood supply, are particularly beneficial for skeletal defects and nonunions where revascularization and integration are critical [2,3]. The restoration of hand and forearm bones, in particular, represents a demanding and precise approach. Besides the considerations about the structural characteristics of the graft and the mechanical demands of the defect, the match of size and shape between donor and defect reflects an integral part for success [4,5]. However, there is a lack of established measurement modalities for determining the ideal size and shape of the vascularized bone graft [6,7]. Visual estimation, which is limited by individual surgeons’ experience and ability, remains the most common method. Furthermore, unstable templates made out of bone wax have not achieved the desired outcome, as their fragility compromises both surgical precision and durability during reconstruction [8,9].

Over recent years, advancements in medical technology have opened new avenues for innovation [10]. Among these, three-dimensional (3D) printing technology has rapidly gained traction and clinical acceptance across multiple domains of reconstructive surgery [9,11,12]. Its integration into clinical practice offers unprecedented surgical tools for precision, customization and meticulous planning [13,14,15]. Preoperative 3D simulations, that are in accordance with CT scans, can objectively quantify exact individual parameters. These personalized simulations are then used to create 3D-printed templates for intraoperative use. Although the use of 3D-printed templates in reconstructive surgery is not entirely new, there is currently no analysis evaluating their impact on vascularized bone reconstruction [16,17,18]. This technology may enable a more accurate shaping of the bone graft and a more precise flap inset, thereby improving clinical outcomes. Moreover, the ability to preoperatively simulate and refine the graft shape in line with each patient’s unique anatomy could lead to better vascular perfusion and structural stability of the graft [19].

We have therefore integrated this innovative technology into the reconstruction of vascularized hand and forearm bones. The present study describes the implementation of 3D simulation and printing processes in clinical practice. Furthermore, we aim to evaluate the technology’s applicability, accuracy and clinical limitations.

## 2. Materials and Methods

This prospective pilot study was conducted in accordance with the local ethical standards and the Helsinki Declaration for Ethical Treatment of Human Subjects, its later amendments and comparable ethical standards. This study was approved by the ethics committee of the local medical faculty (EK no. 1093/2023), and appropriate informed consent was obtained from all the patients. Between June 2019 and June 2024, patients undergoing hand or forearm bone reconstruction were considered for inclusion. The general study inclusion criteria were as follows: vascularized bone graft for hand or forearm bone reconstruction and usage of 3D-printed templates. The patient demographics, including age, sex, follow-up and indication as well as surgical data, were analyzed. Additionally, all the patients underwent preoperative CT scans of the hand, wrist and forearm (Siemens Healthineers, Erlangen, Germany).

### 2.1. Three-Dimensional Simulation

**Figure 1:**
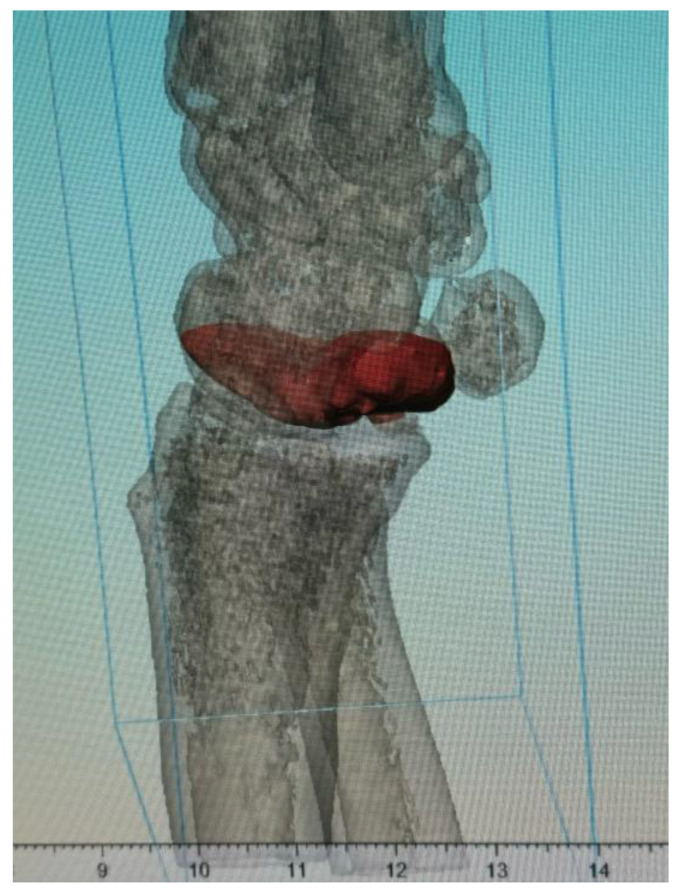
Three-dimensional simulation of the match between the virtually created bone graft (red) and defect. The simulation incorporates the actual characteristics, which were based on the CT scan of the patient.

The CT scan data were imported into 3D conversion software (Mimics Medical, Version 24.0, Materialise, Technologielaan 15, Leuven, Belgium) to facilitate virtual planning and modeling. The simulation and design process were executed using specific software (Materialise 3-matic, Version 18.0, Materialise, Technologielaan 15, Leuven, Belgium). When a CT scan of the contralateral (unaffected) side was available, the corresponding bone structure was virtually mirrored along the vertical axis and incorporated into the affected site to match anatomical symmetry (Figure 1). This method allowed for an accurate duplication of the patient’s unique anatomy, enhancing precision in defect reconstruction.

**Figure 2:**
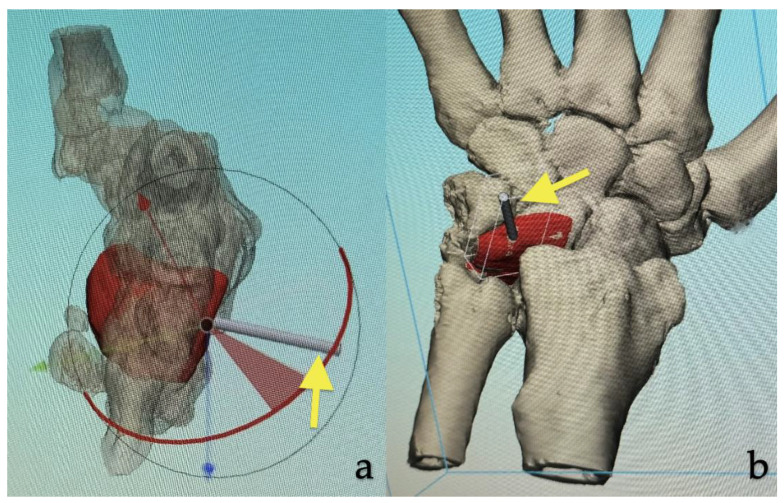
Three-dimensional simulation of the already shaped virtually created bone graft (red). (**a**) A handlebar (yellow arrow) was added at the future entry point of the vascular pedicle. (**b**) This should facilitate handling during surgery.

Two hand surgeons collaboratively refined the virtual bone model to accommodate the specific anatomical characteristics of the donor site. The modifications included adjustments to the bone contour and dimensions, ensuring optimal fit and alignment with the recipient site. If a corresponding contralateral CT scan was not available, a fully virtual bone model was crafted to match the defect’s specific characteristics. Additional considerations in the 3D simulation included identifying the thickness and orientation of any cartilage component within the bone graft, as well as the positioning and orientation of the vascular pedicle. To aid in intraoperative handling, a handlebar feature was added at the planned entry point of the vascular pedicle (Figure 2), ensuring ease of manipulation and precise placement during surgery.

### 2.2. Three-Dimensional Printing

After the creation of the individual designed bone simulation, it was approved for the 3D printing process. The object then gradually and precisely took shape as each thin layer was added according to the simulation. All the prints were performed with a Formlabs 3BL SLA Printer (Funkhaus Berlin, Germany). Medical-approved and sterilizable resin (Formlabs BioMed Clear Resin, Funkhaus Berlin, Germany) was used as the printing material. After 3D printing, the printed templates required washing to remove the residual resin and were cured with an appropriate light (Formlabs Form Cure L, Funkhaus Berlin, Germany). Before sterilization, the template was inspected and verified from the operating surgeon.

### 2.3. Intraoperative Application

For the reconstruction of all the included bone defects, a medial femoral condyle (MFC) flap was utilized. The procedure was executed in a two-team approach, involving both a trauma hand surgeon and a reconstructive microsurgeon. The trauma hand surgeon was responsible for preparing the recipient site, which included osteosynthesis and assessing the viability of the surrounding bone tissue. In cases where avascular bone segments were identified, the 3D template was employed to precisely delineate the resection margins, ensuring that only compromised bone was removed. The 3D template also served as a placeholder, allowing for an exact determination of the osteotomy plane, which helped to guide the orientation of the MFC flap.

**Figure 3:**
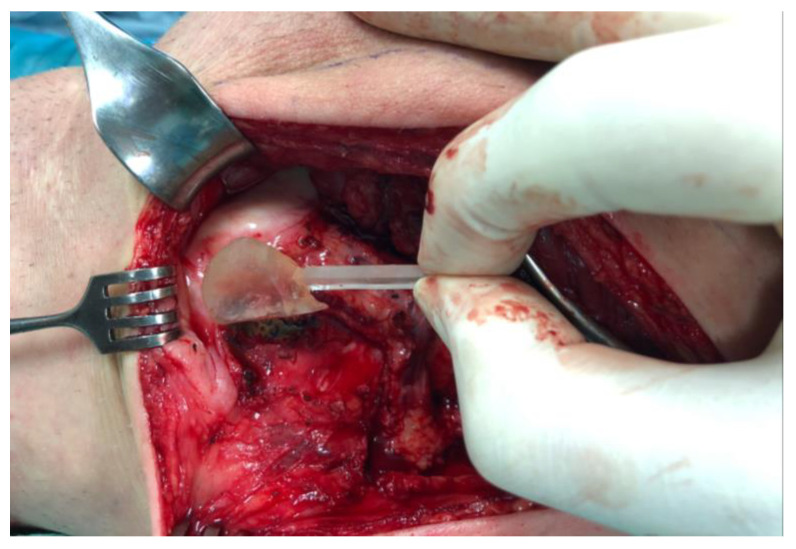
Intraoperative use of the 3D-printed template enabled precise elevation of the MFC flap, taking into account the curvature of the condyle and the orientation of the vascular pedicle.

The MFC flap was elevated following the technique described by Bürger et al. [20], with adjustments made according to each patient’s body conditions. Depending on the specific defect characteristics, the MFC flap could be harvested as either an osteochondral or cortico-cancellous flap. Once the recipient site was fully prepared, the 3D template was again utilized to verify the dimensions, shape and required composition of the MFC flap. Using the template as a guide, the optimal position for flap harvest was marked with respect to the curvature of the condyle and the orientation of the vascular pedicle (Figure 3).

**Figure 4:**
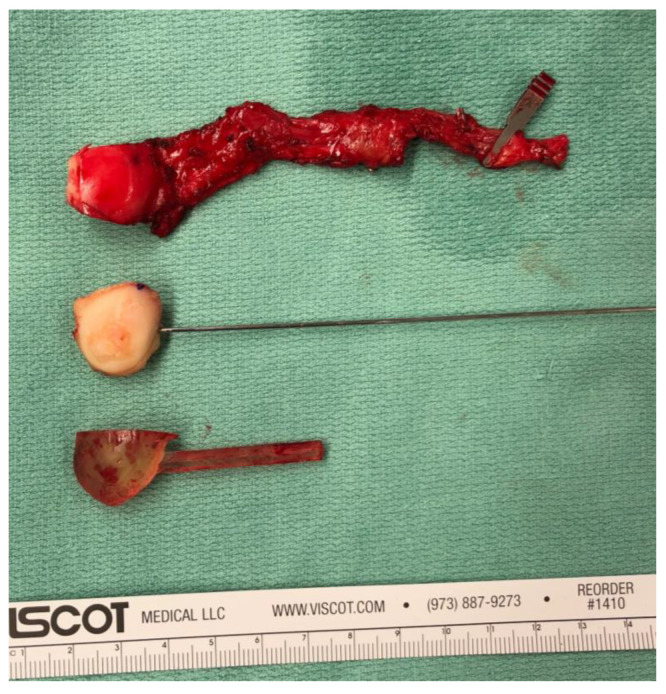
Intraoperative comparison of the 3D-printed template, the avascular bone resectate and the actual MFC flap (elevated according to the 3D-printed model).

To ensure a precise fit within the defect, minor adjustments in the flap size and smoothing of the flap edges were performed as needed before inset. Following flap positioning, osteosynthesis was completed to stabilize the construct, and microvascular anastomosis was carried out to restore blood flow to the graft (Figure 4).

## 3. Results

A total of six patients (five male) underwent vascularized bone reconstruction assisted with 3D technology. Three patients received an osteochondral and three patients received a cortico-cancellous MFC flap for reconstruction. The mean age at the date of surgery was 38 years (range 19–62 years), and the average follow-up time was 15 months (range 5–24 months). The average surgical duration was 310 min (range 251–427 min). Indications for bone reconstruction were avascular necrosis of the carpal bones, a metacarpal defect after tumor resection or pseudoarthrosis after a fractured ulna and are summarized in Table 1. The average dimensions of the defect were 2.2 × 1 × 1.1 cm (range 4.3 − 1 × 1.2–0.5 × 1.6 − 1 cm) and the average dimensions of the used templates were 2.9 × 1.3 × 1.3 cm (range 6 − 3 × 1.7–0.8 × 1.7 − 0.8 cm). No flap loss or major complications were observed. According to the postoperative CT scans, all the MFC flaps were correctly placed with no signs of avascularity. All the patients reported an improved range of motion and reduced pain. Two patients experienced moderate pain at the donor site during physical activity.

### 3.1. Clinical Case

#### 3.1.1. Case 1

**Figure 5:**
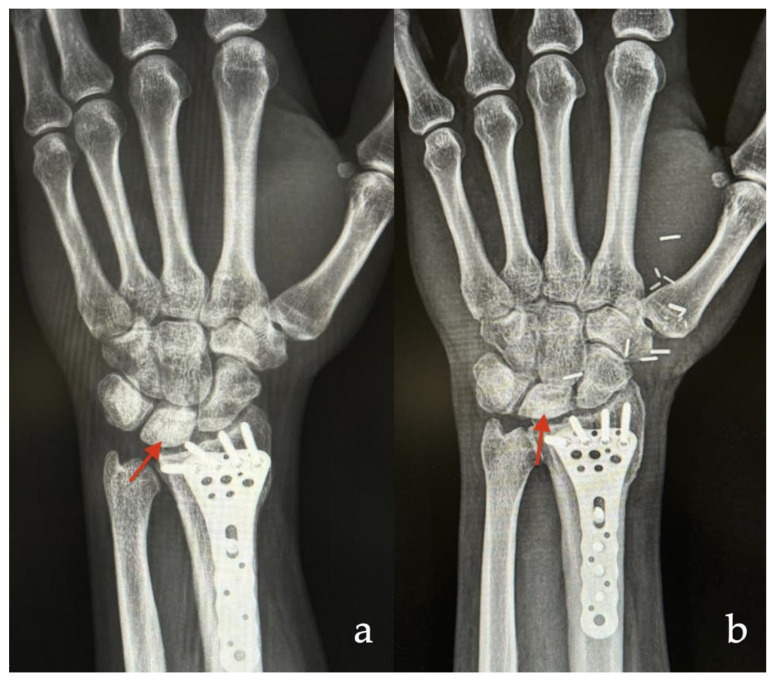
Male patient who underwent vascularized bone reconstruction due to Kienböck’s disease on the left hand. (**a**) Preoperative X-ray—recurrence of partial avascular necrosis of the Lunate (red arrow) two years after radial shortening. (**b**) Postoperative X-ray—one year after carpal bone reconstruction with free MFC bone flap (red arrow) assisted with 3D technology.

A 40-year-old male patient underwent vascularized bone reconstruction due to Kienböck’s disease on the left hand. Although he received radial shortening two years before, recurrence of partial avascular necrosis of the Lunate appeared (Figure 4). Hence, we decided to use a free MFC flap for vascularized bone reconstruction. Incorporating individual defect characteristics, a 3D simulation and its corresponding template were conducted for the patient. The MFC flap with dimensions of 1 × 0.5 × 1 cm was raised in a cortico-cancellous fashion according to the shape of the template. Then, the flap was fixated with K-wires into the carpal defect. Thereafter, microanastomosis (end to side) to the radial artery and accompanying vein (using vein coupler) was performed. At the one-year follow-up, our patient showed an increased range of motion without any pain (Figure 5).

#### 3.1.2. Case 2

**Figure 6:**
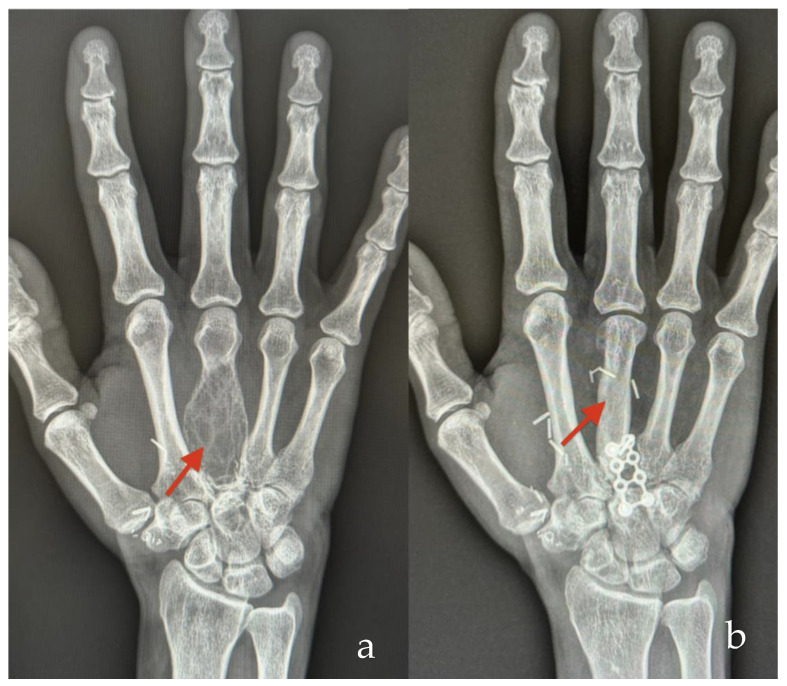
Female patient who underwent vascularized bone reconstruction due to resection of giant cell tumor (red arrow) of her right hand. (**a**) Preoperative X-ray—recurrence of giant cell tumor of the metacarpal bone. (**b**) Postoperative X-ray—one year after metacarpal bone reconstruction with free MFC bone flap (red arrow) assisted with 3D technology.

A 47-year-old woman presented with a recurrent, rapidly growing giant cell tumor (GCT) on the dorsum of her right hand, two years after an initial resection of the GCT on her third metacarpal (Figure 6). Following wide excision of the tumor and removal of the metacarpal (except for the head), the personalized 3D-printed template was used to precisely design a replacement using an MFC cortico-cancellous flap. This model enabled accurate flap shaping and planning for fixation with miniplates. During surgery, the MFC flap was harvested, matched to the template and secured with locking plates. Thereafter, microanastomosis (end to side) to the radial artery and accompanying vein (using vein coupler) was performed. At the one-year follow-up, the third metacarpal had been successfully restored to its normal length with complete bony union achieved and no evidence of tumor recurrence (Figure 6).

## 4. Discussion

Vascularized bone reconstruction represents a promising treatment option for patients with a variety of complex bone pathologies [21,22]. In recent years, surgical aspects of flap harvesting and microsurgery have become routine in reconstructive surgery [23,24]. However, there is a lack of supporting modalities for objective and accurate bone shaping, which remains an ongoing challenge of this procedure [25]. Therefore, the primary aim of this study was to improve the accuracy of bone graft shaping by using individually created 3D-printed templates. Furthermore, this study aims to describe the process of implementing and the clinical application of 3D simulation and printing technology.

This innovative technology has emerged as a supportive tool for clinical purposes, particularly in surgical planning [26,27,28,29]. Beginning with the simulation process, a virtual bone graft was created in accordance with the imported CT scan data. Since a standard preoperative CT scan was already conducted, no additional appointments or investigations were required for the patients. The presence of an experienced hand surgeon during the simulation process ensured an optimal fusion of virtual and actual surgical requirements. Specific characteristics and dimensions of the defect were analyzed and incorporated into the simulation accordingly. When no suitable contralateral model was available, a fully virtual creation of the required bone suiting the patients’ posture was crafted. This precondition remains challenging for intraoperative application and reflects a certain kind of limitation. With regards to our patient cohort, we observed one patient with bilateral advanced Kienböck’s disease presented a unique challenge. In this case, the fully virtual template did not optimally fit the defect. While the shape was accurate, the size was too large for the actual defect. This observation is consistent with our overall findings regarding the size match between the defect and the corresponding template. Due to the pilot study design, it should be classified as practical experience. Furthermore, it is more feasible to marginally reduce the size of the bone graft after harvest than to enlarge it.

Another essential aspect is the location and orientation of the pedicle and its relation to the defect. For all included bone defects, the free MFC flap was used for reconstruction.

Due to the relatively constant anatomy of this flap, the orientation of the pedicle and the virtually added handlebar corresponded in most cases [30]. Even then, when there was a variation in the supplying vessel, resulting in intraoperative adjustments of the flap inset, the haptic template can enhance imagination of the adapted inset. Although we exclusively used the free MFC flap, other flaps such as the vascularized iliac-crest flap or lateral femoral condyle flap are potential alternatives for bone reconstruction [31,32]. In addition to the pedicle’s location, the composition of the bone graft, including surface conformity, is crucial for planning. The application of 3D simulation incorporated these considerations and allowed us to shift from intraoperative visual estimation and personal experiences to precise preoperative planning.

For intraoperative use, the 3D-printed template first served to conform the defect dimensions, guiding the resection of any avascular bone segments to match the template’s customized shape. After this initial alignment, the template was positioned as a placeholder, enabling more accurate placement of the miniplates for osteosynthesis, thereby reducing the operative time and minimizing the handling of the vascularized bone graft. During the flap elevation, the template was applied to mark the precise resection area on the surface of the medial femoral condyle (MFC) flap, respecting its natural curvature and the orientation of the vascular pedicle. Final adjustments to the bone flap, such as slight adaptations and contour refinements, were completed without an oscillating saw, allowing for the preservation and precise shaping of the graft to match both the template and defect requirements. As both “surgical teams” were able to refer to the 3D template, coordination and simultaneous performance were optimized, resulting in a more efficient workflow and a reduced overall surgical duration. Compared to our previously performed cases, the surgical duration was notably shorter when using the template. However, further research is needed to confirm this finding with greater certainty.

The use of this supportive technology did not alter the indication for vascularized bone reconstruction or affect patient selection criteria. However, depending on the bone being reconstructed, the flap requirements varied significantly. Bone flaps for larger, cylindrical structures, such as the metacarpals or forearm bones, were relatively straightforward to shape, while carpal bone reconstruction demanded more intricate contouring due to their compact size and complex curvature. Nonetheless, the use of patient-specific templates enhanced the congruence between the donor bone and defect site, optimizing the fit and contour for each anatomical structure, regardless of complexity.

### Limitations

While this study highlights the potential benefits of personalized 3D-printed templates in vascularized bone reconstruction, several limitations warrant discussion. First, the small sample size of six patients limits the validity of our findings. Although our results suggest that 3D-printed templates may improve surgical precision and outcomes, a larger cohort is needed to validate these preliminary findings and to establish statistical significance. With this in mind, a prospective randomized controlled trial (RCT) should be conducted in future research to enable a detailed comparison and address the limitations of this pilot study. Based on preliminary estimates, such a trial would require approximately 30–50 patients per group to achieve 80–90% statistical power at a significance level of 0.05. Furthermore, the short average follow-up period of 15 months restricts our ability to assess the long-term outcomes and potential complications associated with the use of this technology in this setting. Longer follow-up is essential to fully understand the durability of these reconstructions, as well as to monitor for any late-onset complications, such as graft avascularity or structural failure.

In addition, implementing 3D printing technology in clinical practice presents significant challenges. The use of 3D-printed templates requires access to specialized equipment and technical expertise, both of which may be limited in certain clinical settings. Establishing a workflow that incorporates advanced imaging, software-based design and template production also demands considerable time and resource investment. Opportunely, the Department of Oral and Maxillofacial Surgery at our hospital founded a 3D simulation and printing laboratory in 2011. Since its inception, practical experiences, evolving trends and technical developments have contributed to the growth of this lab into an established and recognized institute at our hospital. Beginning with personalized templates for vascularized bone reconstruction in 2019, various simulation and printing processes had already been put in place and were selectively adopted for this specific purpose. As a result, 3D prints were produced at low costs and with relative speed. However, future studies should explore the cost-effectiveness, reproducibility and scalability of using 3D-printed templates in vascularized bone reconstruction to better inform the feasibility of widespread clinical implementation.

## 5. Conclusions

The present study describes the application and implementation of 3D simulation and printing technology for vascularized bone reconstruction. Patient-specific 3D-printed templates, based on individual 3D simulations tailored to defect characteristics, were used to ensure accurate flap elevation, shaping and inset. Our evaluation of the clinical application revealed improvements in both preoperative planning and intraoperative performance. Although the implementation of this technology required certain resources and time, we have successfully integrated it into our standard routine and highly recommend its use for vascularized bone reconstruction.

## Figures and Tables

**Table 1:** Demographical data.

Patient	Age (yr)	Sex	Location	Indication	Composition MFC Flap	Time of Follow-Up (Months)
1	38	M	Scaphoid	Nonunion (avascular necrosis)	Osteochondral	24
2	19	M	Scaphoid	Nonunion (avascular necrosis)	Osteochondral	12
3	20	M	Lunate	Kienböck’s disease	Osteochondral	5
4	40	M	Lunate	Kienböck’s disease	Cortico-cancellous	18
5	47	F	Metacarpal bone	Resection of giant cell tumor	Cortico-cancellous	18
6	62	M	Ulna	Nonunion (post-traumatic)	Cortico-cancellous	12

F, female; M, male; and yr, year.

## Data Availability

The data are contained within this article.
